# Supplementary figures and images for: Short-term wind power forecasting through stacked and bi directional LSTM techniques
Source: PeerJ Comput Sci. 2024 Mar 29;10:e1949. doi: 10.7717/peerj-cs.1949 (PMC11042035; doi:10.7717/peerj-cs.1949)

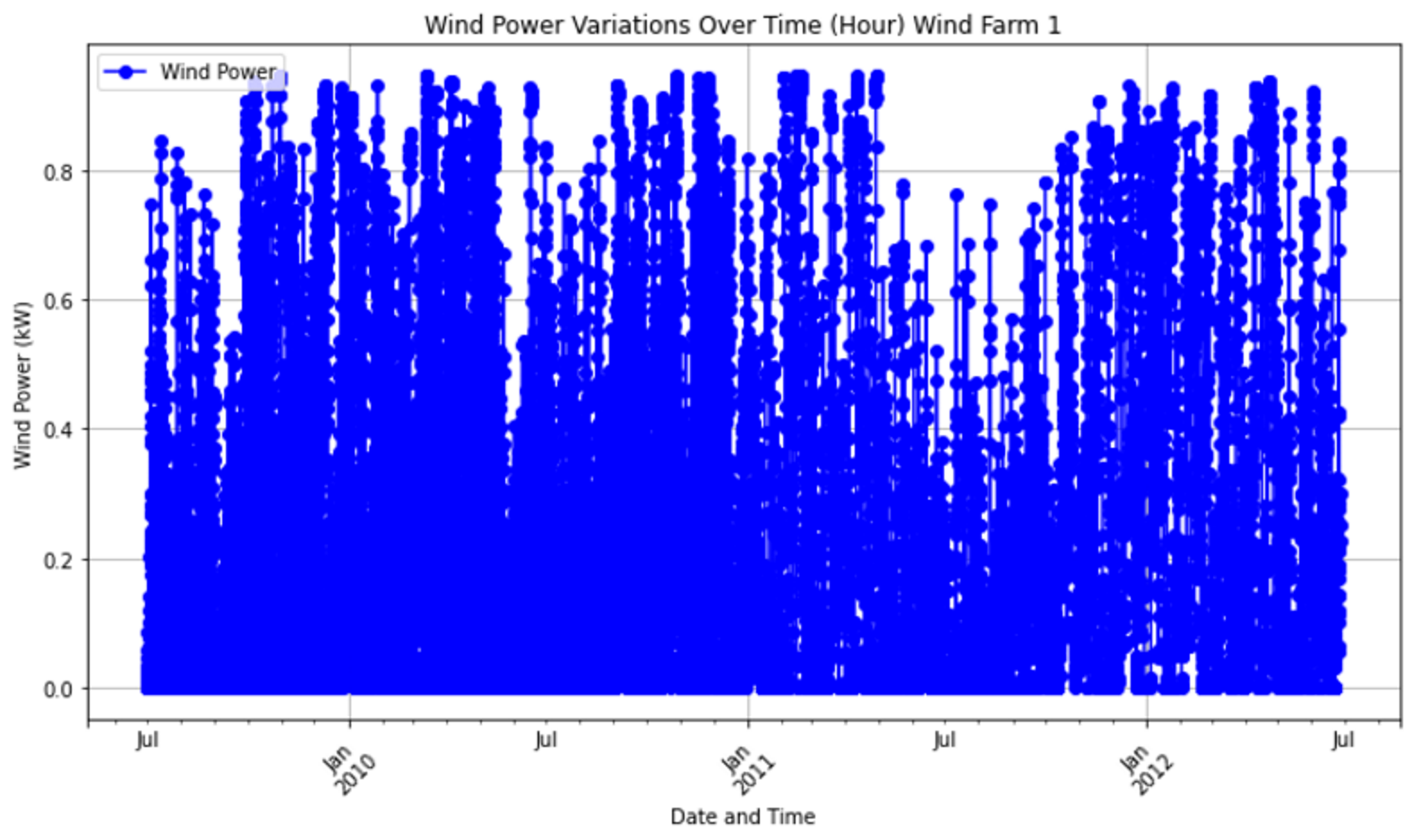

Supplement: Supplemental Information 1 [file peerj-cs-10-1949-s001.png]

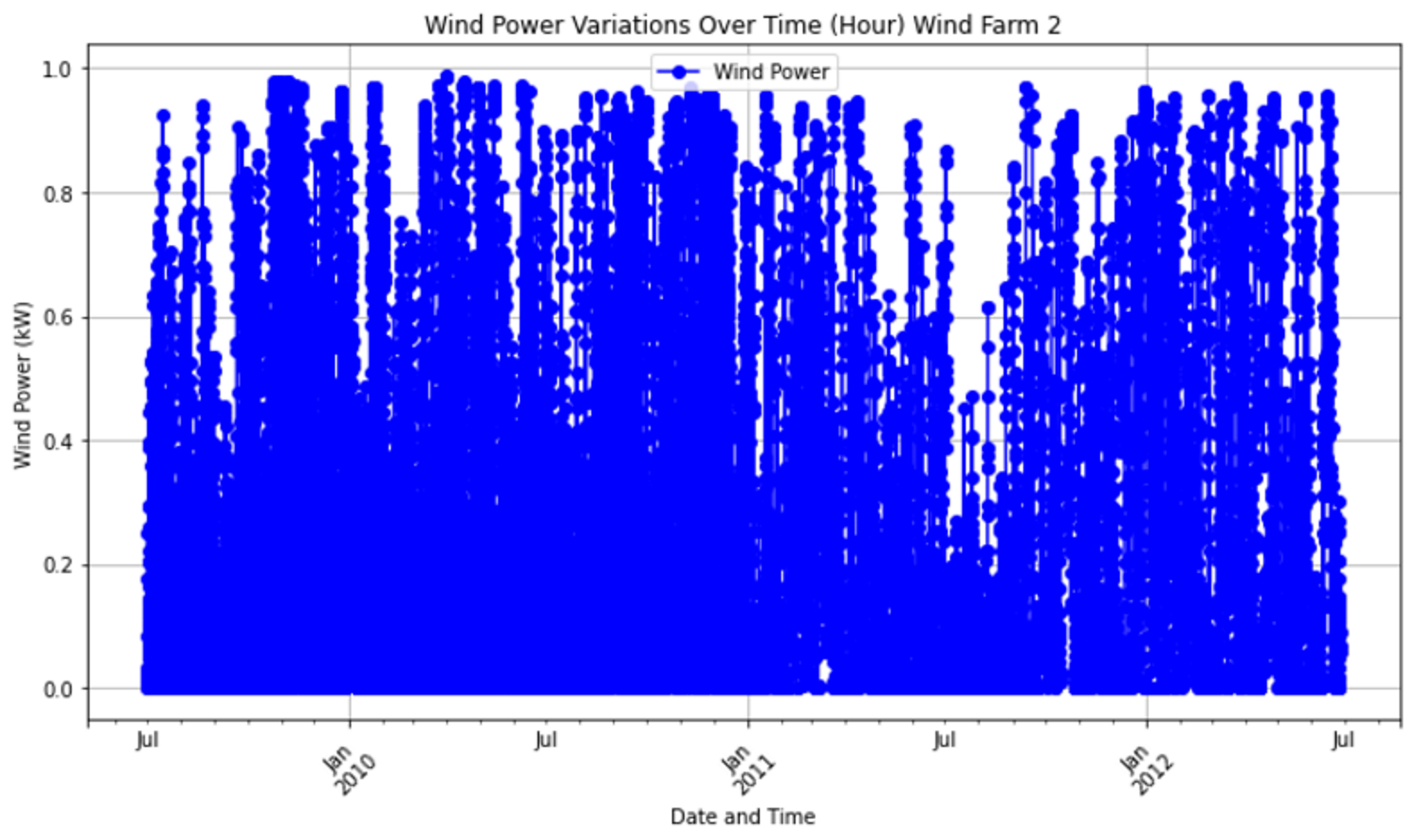

Supplement: Supplemental Information 2 [file peerj-cs-10-1949-s002.png]

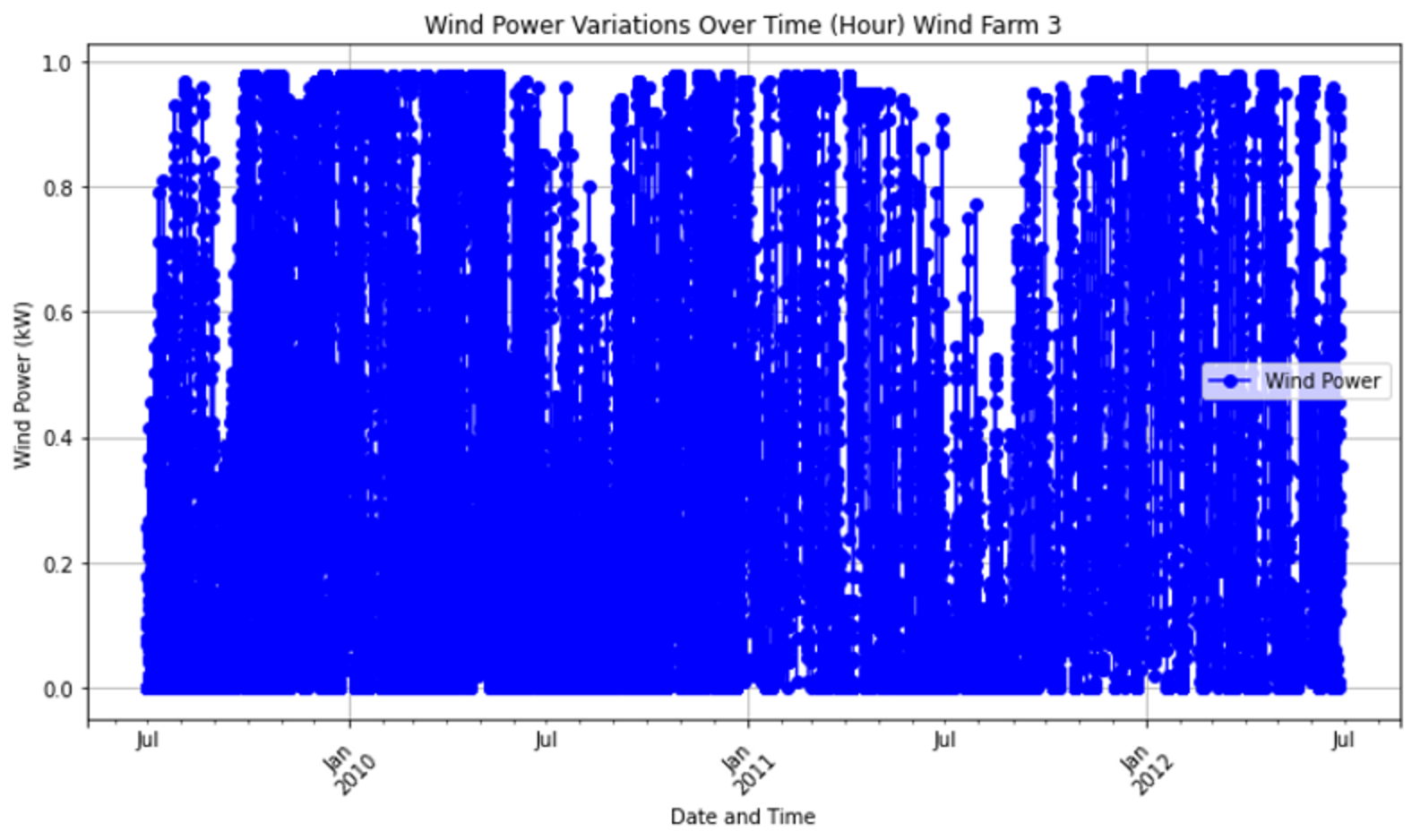

Supplement: Supplemental Information 3 [file peerj-cs-10-1949-s003.png]

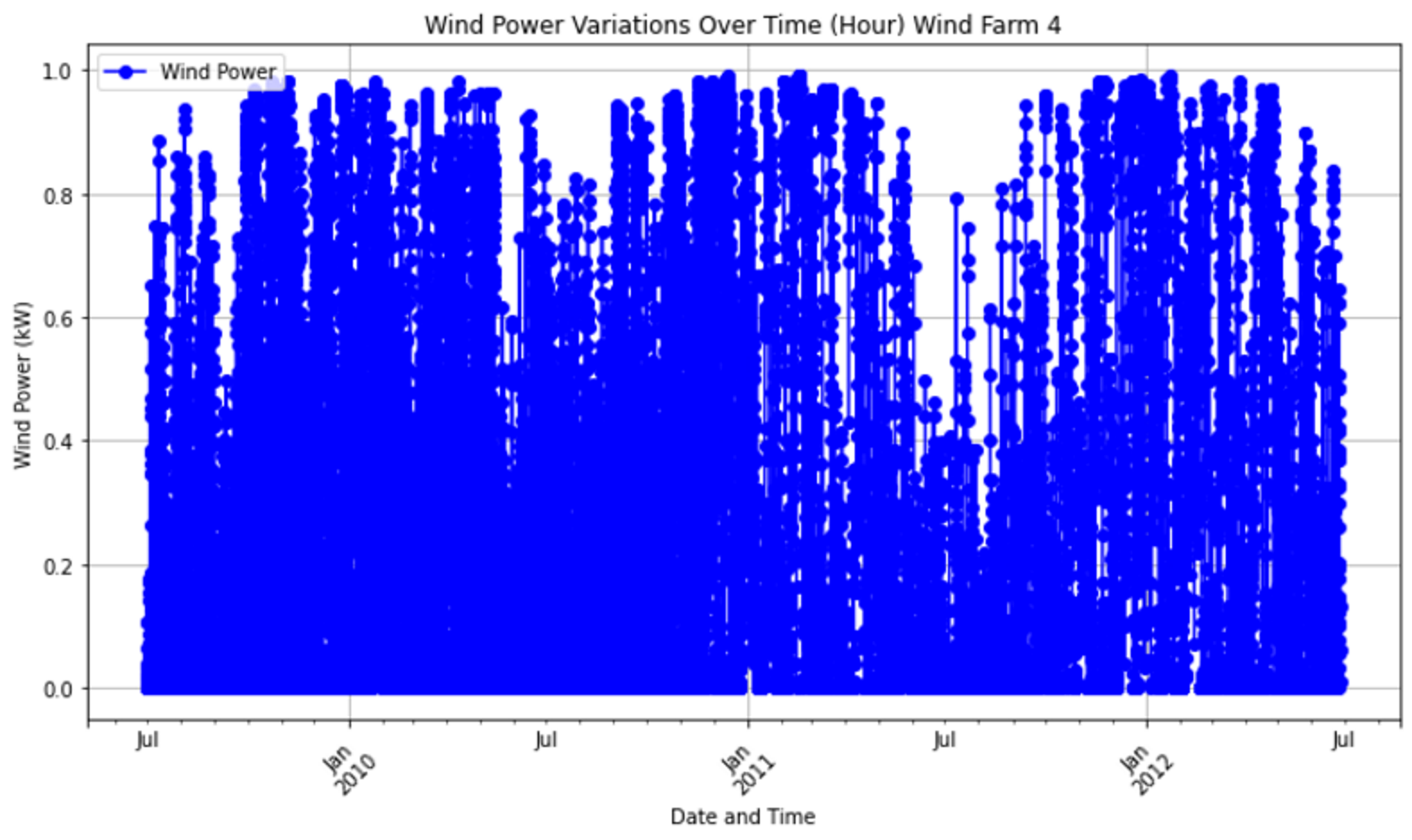

Supplement: Supplemental Information 4 [file peerj-cs-10-1949-s004.png]

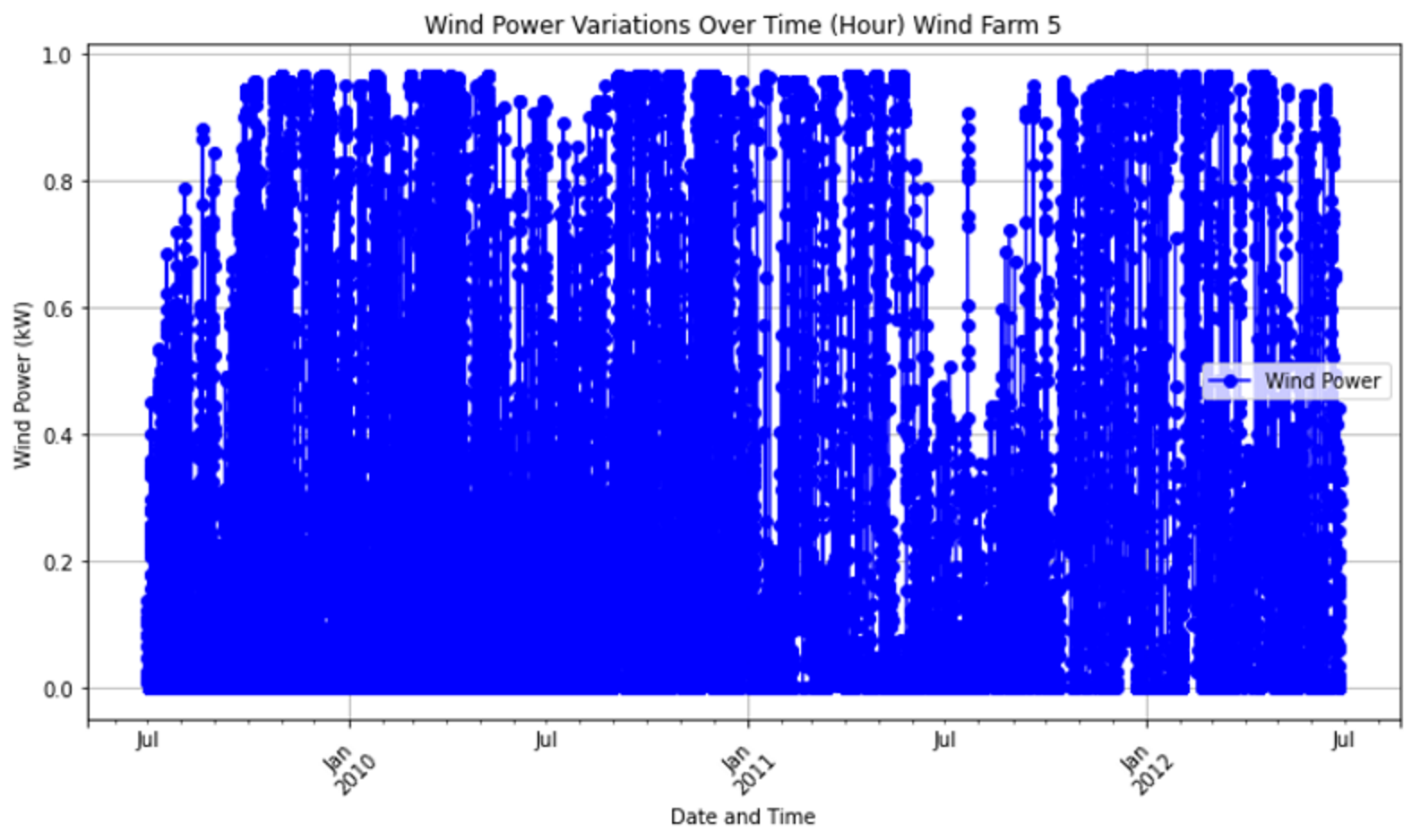

Supplement: Supplemental Information 5 [file peerj-cs-10-1949-s005.png]

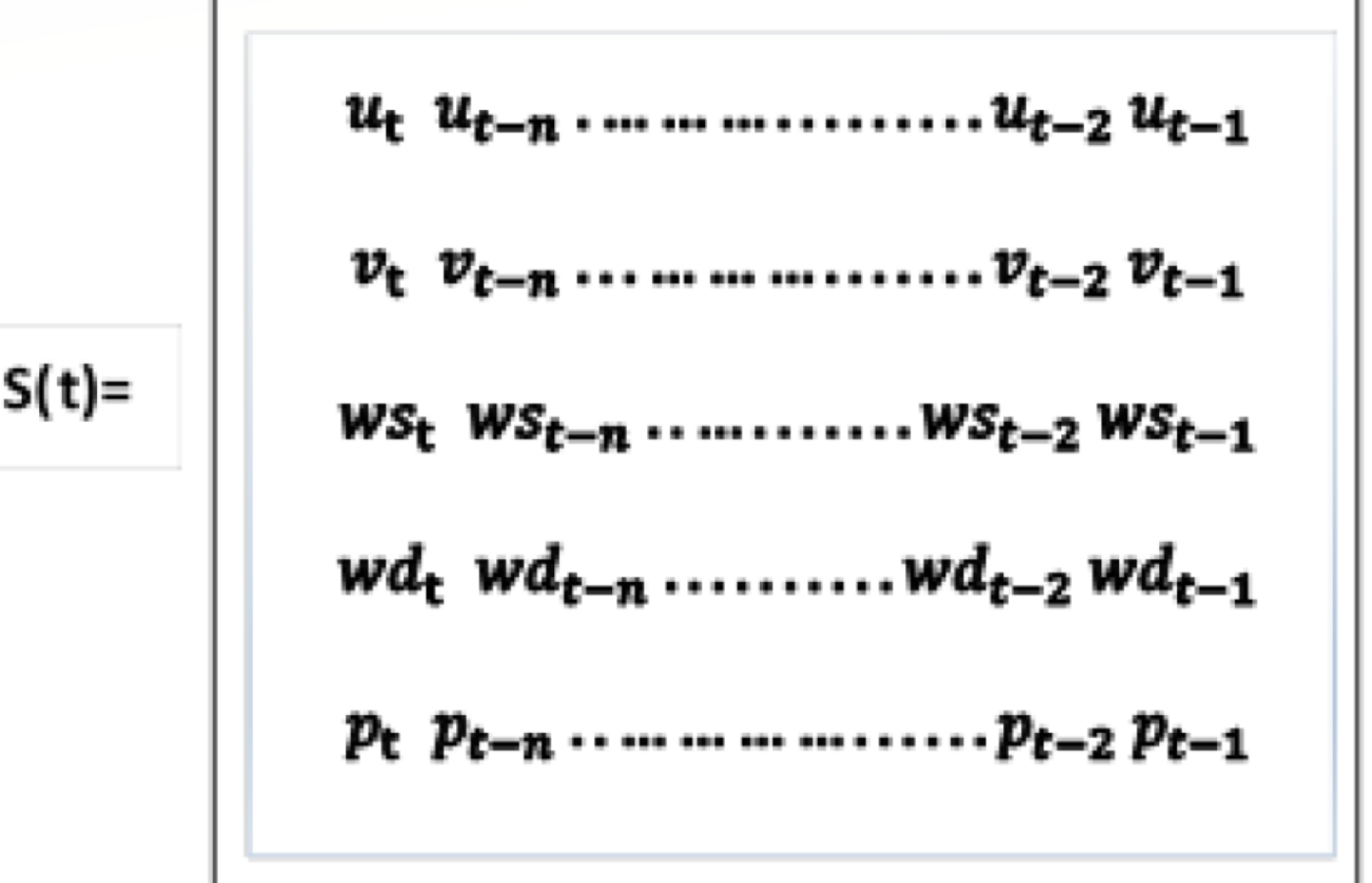

Supplement: Supplemental Information 6 [file peerj-cs-10-1949-s006.png]

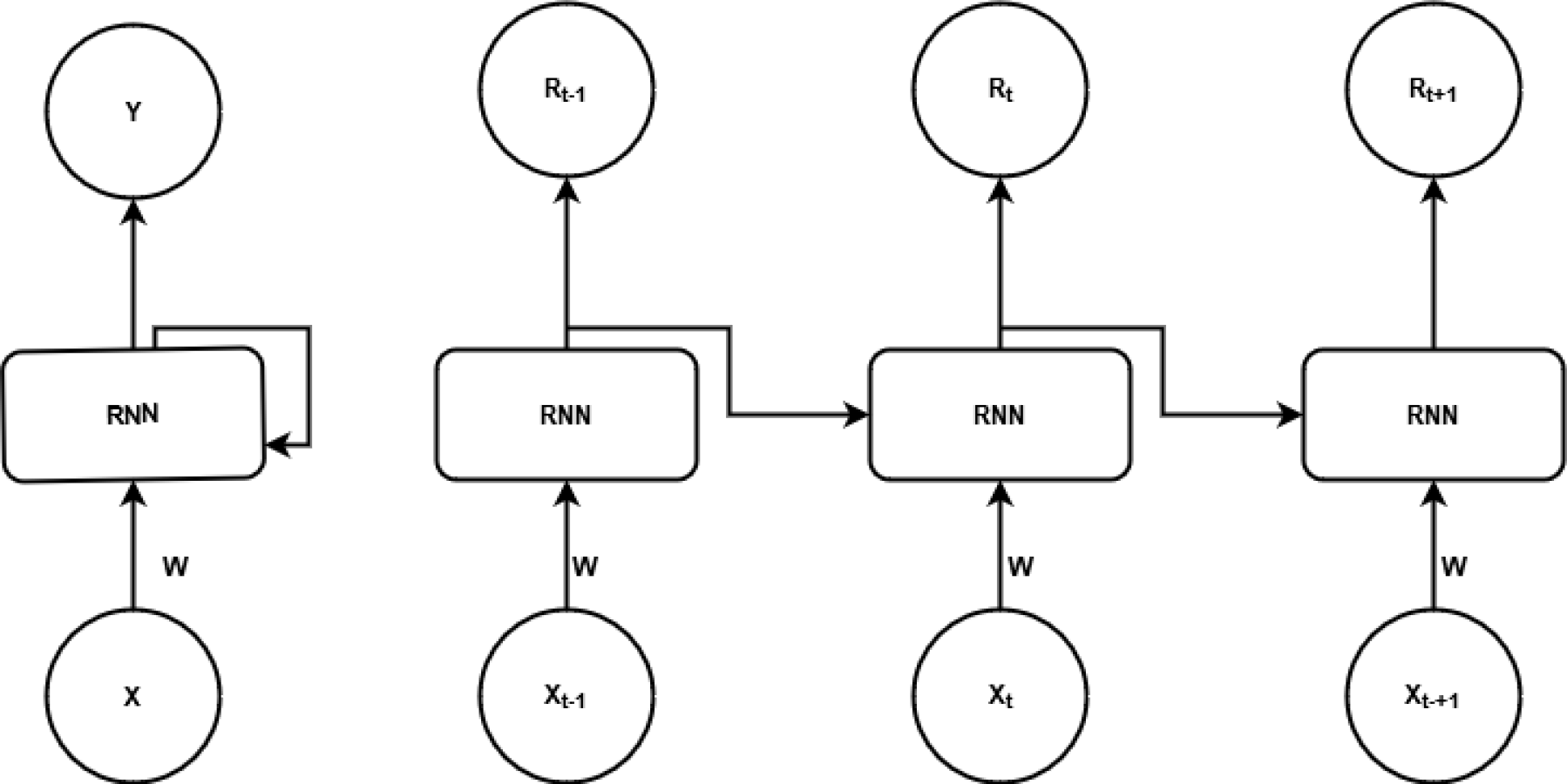

Supplement: Supplemental Information 7 [file peerj-cs-10-1949-s007.png]

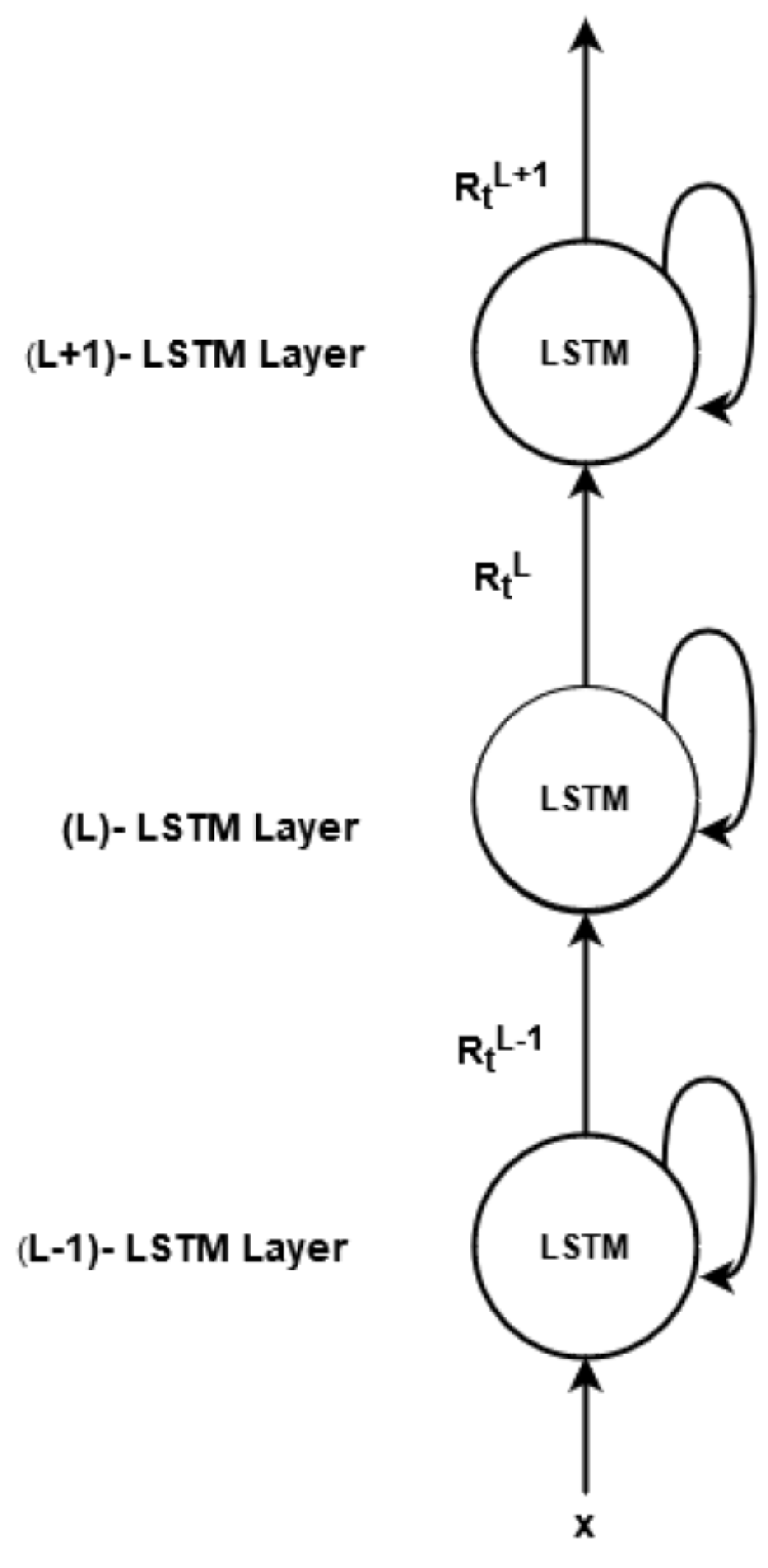

Supplement: Supplemental Information 8 [file peerj-cs-10-1949-s008.png]

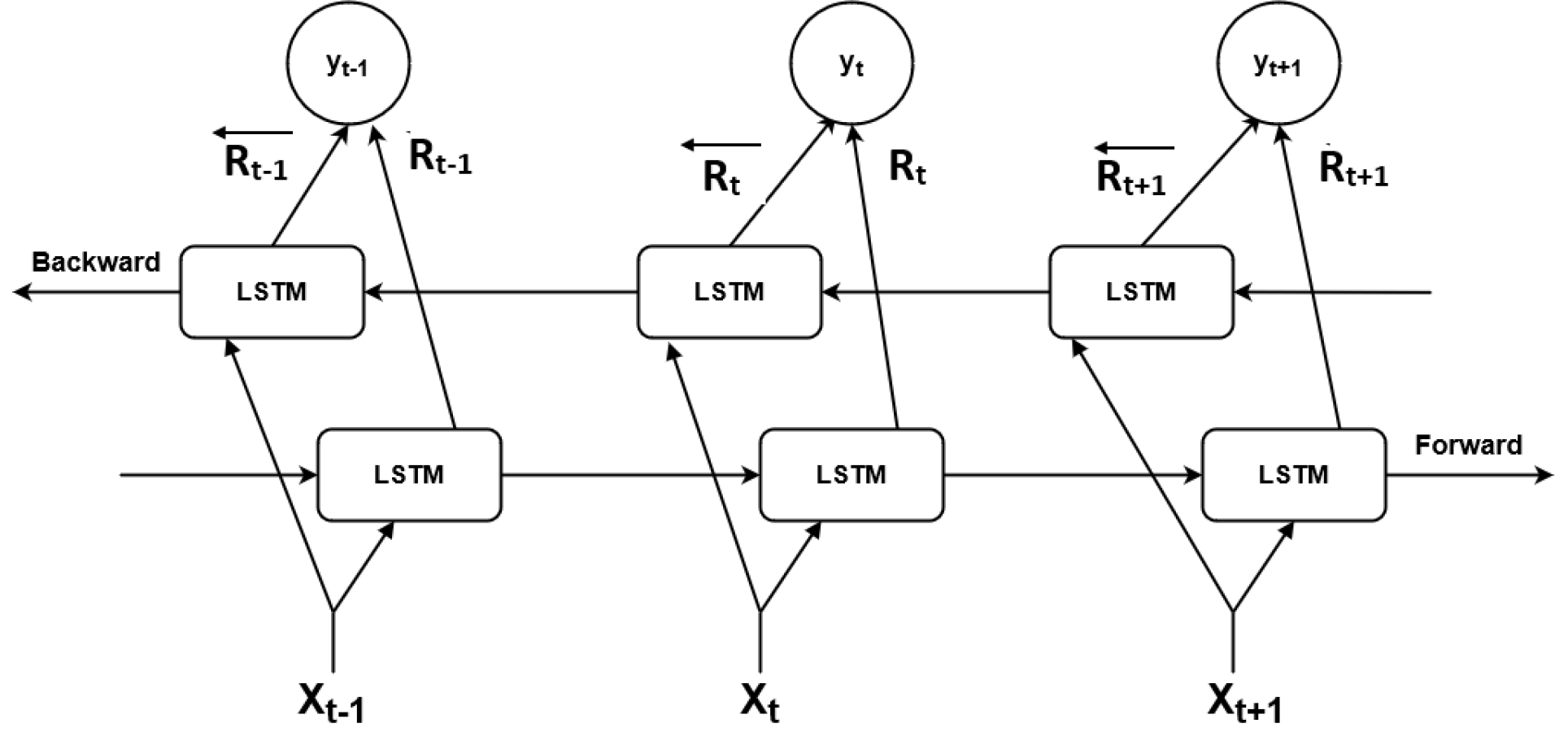

Supplement: Supplemental Information 9 [file peerj-cs-10-1949-s009.png]
