# Supplementary material for: Short-term wind power forecasting through stacked and bi directional LSTM techniques
Source: PeerJ Comput Sci. 2024 Mar 29;10:e1949. doi: 10.7717/peerj-cs.1949 (PMC11042035; doi:10.7717/peerj-cs.1949)
Supplement: Supplemental Information 10 [file peerj-cs-10-1949-s010.pdf]

Wind Farm 1 - Birdirectional LSTM

Epochs	Training Loss	Validation Loss	MSE Training loss	MSE Validation Loss
epoch 1	0.0067	0.0042	0.0136	0.0085
epoch 2	0.0041	0.0037	0.0083	0.0075
epoch 3	0.0037	0.0036	0.0074	0.0072
epoch 4	0.0034	0.0046	0.0068	0.0093
epoch 5	0.0033	0.0042	0.0066	0.0085

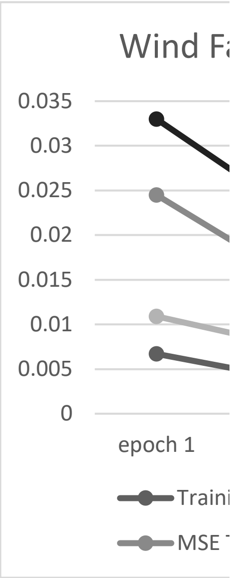

Wind Farm 1 - Stacked LSTM

Epochs	Training Loss	Validation Loss	MSE Training loss	MSE Validation Loss
epoch 1	0.0072	0.0048	0.0145	0.0098
epoch 2	0.0037	0.0039	0.0075	0.0079
epoch 3	0.0032	0.0037	0.0064	0.0074
epoch 4	0.0029	0.0036	0.0059	0.0072
epoch 5	0.0028	0.0035	0.0056	0.007

Wind Farm 2 - Birdirectional LSTM

Epochs	Training Loss	Validation Loss	MSE Training loss	MSE Validation Loss
epoch 1	0.0075	0.0059	0.0151	0.0119
epoch 2	0.0044	0.0057	0.0089	0.0117
epoch 3	0.0039	0.0069	0.0079	0.014
epoch 4	0.0037	0.0054	0.0075	0.0111
epoch 5	0.0035	0.0047	0.0071	0.0096

Wind Farm 2 - Stacked LSTM

Epochs	Training Loss	Validation Loss	MSE Training loss	MSE Validation Loss
epoch 1	0.0084	0.006	0.017	0.0123
epoch 2	0.0038	0.0055	0.0077	0.0113
epoch 3	0.0034	0.0057	0.0069	0.0117
epoch 4	0.0032	0.0046	0.0064	0.0095
epoch 5	0.003	0.0046	0.0061	0.0094

Wind Farm 3 - Bidirectional LSTM

Epochs	Training Loss	Validation Loss	MSE Training loss	MSE Validation Loss
epoch 1	0.0109	0.0146	0.0223	0.0297
epoch 2	0.0069	0.0066	0.014	0.0135
epoch 3	0.0062	0.0067	0.0126	0.0137
epoch 4	0.006	0.0072	0.0121	0.0146
epoch 5	0.0059	0.0065	0.012	0.0134

Wind Farm 3 - Stacked LSTM

Epochs	Training Loss	Validation Loss	MSE Training loss	MSE Validation Loss
epoch 1	0.0111	0.0087	0.0227	0.0178
epoch 2	0.0059	0.007	0.0119	0.0144
epoch 3	0.0053	0.0063	0.0107	0.013
epoch 4	0.005	0.006	0.0101	0.0124
epoch 5	0.0049	0.006	0.0099	0.0124

Wind Farm 4 - Bidirectional LSTM

Epochs	Training Loss	Validation Loss	MSE Training loss	MSE Validation Loss
epoch 1	0.0104	0.0063	0.0211	0.0129
epoch 2	0.0064	0.0054	0.013	0.0111
epoch 3	0.0055	0.0067	0.0111	0.0136
epoch 4	0.0052	0.0052	0.0105	0.0107
epoch 5	0.005	0.005	0.01	0.0102

Wind Farm 4 - Stacked LSTM

Epochs	Training Loss	Validation Loss	MSE Training loss	MSE Validation Loss
epoch 1	0.0103	0.0073	0.0209	0.0149
epoch 2	0.0053	0.0055	0.0106	0.0112
epoch 3	0.0046	0.0051	0.0094	0.0104
epoch 4	0.0043	0.0058	0.0088	0.0117
epoch 5	0.0042	0.0054	0.0084	0.011

Wind Farm 5 - Bidirectional LSTM

Epochs	Training Loss	Validation Loss	MSE Training loss	MSE Validation Loss
epoch 1	0.0097	0.0083	0.0197	0.0171
epoch 2	0.0061	0.0076	0.0123	0.0157
epoch 3	0.0055	0.0127	0.0112	0.026
epoch 4	0.0052	0.0066	0.0106	0.0136
epoch 5	0.005	0.0066	0.0101	0.0136

Wind Farm 5 - Stacked LSTM

Epochs	Training Loss	Validation Loss	MSE Training loss	MSE Validation Loss
epoch 1	0.0101	0.0087	0.0207	0.0178
epoch 2	0.0055	0.01	0.0111	0.0204
epoch 3	0.0048	0.0072	0.0098	0.0149
epoch 4	0.0045	0.007	0.0091	0.0144
epoch 5	0.0044	0.0073	0.0088	0.0149

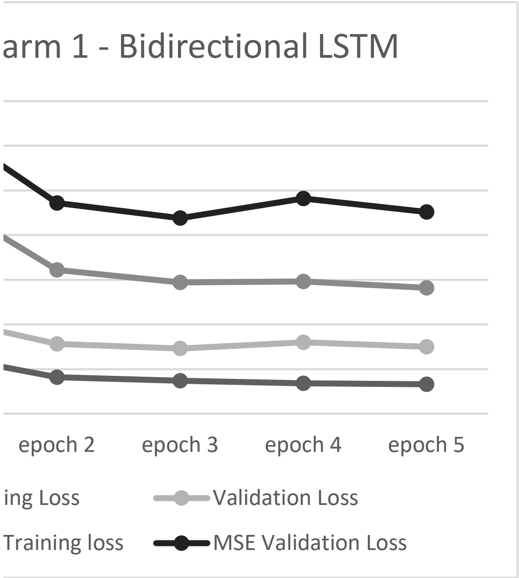
arm 1 - Bidirectional LSTM
epoch 2
epoch 3
epoch 4
epoch 5
ing Loss
Validation Loss
Training loss
MSE Validation Loss

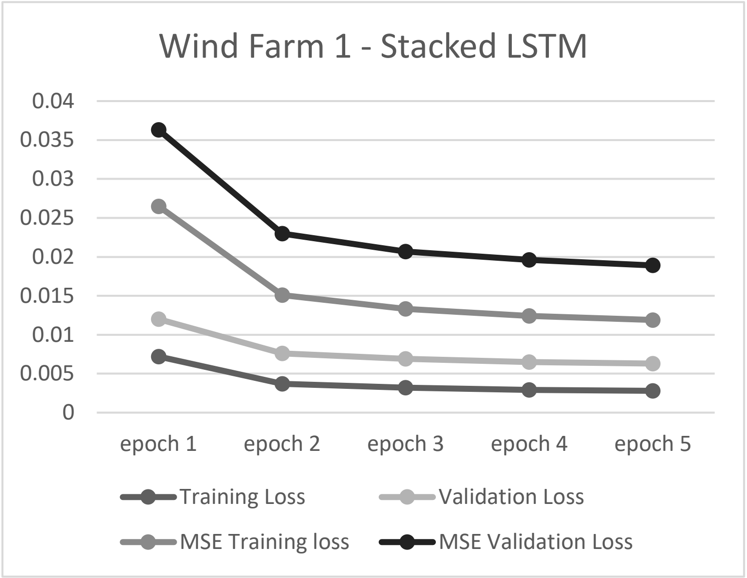
Wind Farm 1 - Stacked LSTM
0.04
0.035
0.03
0.025
0.02
0.015
0.01
0.005
0
epoch 1
epoch 2
epoch 3
epoch 4
epoch 5
Training Loss
Validation Loss
MSE Training loss
MSE Validation Loss
